# Integrated pH-responsive three-phase system: Time- and solvent-efficient biorefining of *Camellia oleifera* waste into multifunctional saponins

**DOI:** 10.1016/j.fochx.2025.102870

**Published:** 2025-08-05

**Authors:** Zhihong Chen, Jinlin Fan, Chao Zhao, Taoyuan Huang, Zhiying Guo, Changyang Qiu, Jiacong Deng

**Affiliations:** a College of Food and Bioengineering, Fujian Polytechnic Normal University, Fuqing 350300, China; b Institute of Jiangxi Oil-tea Camellia, Jiujiang University, Jiujiang 332005, China; c College of Marine Sciences, Fujian Agriculture and Forestry University, Fuzhou 350117, China

**Keywords:** Three-phase partitioning, pH-responsive extraction, Saponins, *Camellia oleifera* waste, Bioemulsifiers

## Abstract

*Camellia oleifera* seed cake (COSC), an agricultural byproduct, offers untapped potential as a biosurfactant source but suffers from inefficient extraction. We developed a pH-responsive platform integrating ultrasound-assisted alkaline three-phase partitioning (*t*-butanol/ammonium sulfate, pH 10) and acid-triggered precipitation (pH 4.5). This achieved 13.5 g saponins/100 g COSC in 30 min (92 % faster than Soxhlet) with 91 % *t*-butanol recovery. Acid-driven purification eliminated organic solvents, attaining 85 % purity versus conventional 70 %. Scalability was validated through pilot-scale trials (22.5–25 L working volume), achieving 12.7 g/100 g yield and 82 % purity. FTIR/HPLC analysis confirmed intact oleanane-type triterpenoids, correlating with superior functionality: critical micelle concentration of 1.0 % (vs. 3.0 % for conventional extract), 14-day emulsion stability, and enhanced bioactivities (DPPH IC_50_ 1.23 g/L; MIC 1.29 g/L). Three breakthroughs were achieved: 1) pH-responsive efficiency-purity synergy, 2) closed-loop solvent reuse, and 3) acetone-free precipitation. This work provides a scalable, sustainable route to valorize agro-industrial residues into high-performance plant-based emulsifiers.

## Highlights

• UATPP-AP extracts 13.5 g/100 g saponins in 30 min (92 % faster).
• Closed-loop *t*-butanol recycling (91 %) with solvent-free crystallization (85 % purity)
• Scalable process maintains 82 % purity, 12.7 g/100 g yield at 22.5–25 L vol.
• Purified saponins achieve 1.0 % CMC (vs 3.0 % conventional) & 14-day stable emulsions.
• pH-responsive purification retains dual antioxidant/antimicrobial saponins.

## 1 Introduction

Saponins constitute a structurally diverse family of glycosides characterized by their inherent amphiphilicity, arising from the covalent linkage between hydrophilic carbohydrate moieties and hydrophobic aglycone scaffolds (predominantly triterpenoid or steroidal frameworks) (Timilsena et al., 2023). This molecular duality drives their exceptional emulsification performance, enabling effective oil-water interface stabilization at reduced dosage levels coupled with superior resistance to droplet coalescence relative to synthetic counterparts (Zhang et al., 2023). Currently, saponins have been used industrially as foamers and emulsifiers in beer and soft drinks (Guclu-Ustundag & Mazza, 2007), as solubilizing agents for vitamins and minerals in food additives (Jenkins & Atwal, 1994). Saponins also exhibit a variety of biological activities linking to human health benefits (Xia et al., 2024; Xiao et al., 2025). Hence, the naturally occurring saponins that not only possess remarkable interfacial activity but exhibit nontrivial bioactivities, are highly attractive bifunctional emulsifiers for the formulation of colloidal multiphase food systems.

However, the extraction of plant-derived saponins remains technically challenging due to their amphiphilic structure. These compounds exhibit sharply contrasting solubility behaviors: insoluble in nonpolar solvents (e.g., chloroform), limited solubility in cold water/short-chain alcohols (e.g., ethanol), partial dissolution in warm aprotic solvents (e.g., ethyl acetate), and complete solubility only in heated hydroxyl-rich solutions (e.g., hydroalcoholic mixtures) (Yu & He, 2018a). Consequently, the extraction methods for saponins are primarily confined to hot water extraction and ethanol water extraction. Nevertheless, both water-based and ethanol-water extraction methods exhibit similar drawbacks particularly regarding high solvent consumption, lengthy extraction durations, and substantial energy requirements, which makes extraction on an industrial scale difficult (Chi et al., 2023). Besides, In terms of purification methods, there are also many drawbacks. The acetone precipitation method is widely used for saponins purification, however, the addition of acetone is toxic and can cause severe environmental pollution (Yu & He, 2018b). Other processes such as column chromatography, molecularly imprinted polymers and flocculation have disadvantages of complex, high cost and large investment (Cheok et al., 2014). Consequently, it is vitally important to develop an efficient, cost-effective and green technique to overcome those drawbacks in saponins extraction and purification.

The pH-dependent solubility technique, utilizing sequential alkaline dissolution and acidic phase separation, provides a cost-effective strategy for fractionating complex matrices. This methodology has demonstrated remarkable versatility in food science applications, enabling selective recovery of polyphenolic compounds (e.g., anthocyanins), flavonoid glycosides, plant-derived proteins (60 %–80 % recovery rate), and amphoteric bioactive constituents through controlled isoelectric precipitation (Jiang et al., 2021; Li et al., 2014; Zhang et al., 2020). The two-stage separation includes alkaline extraction and acid purification. It is noteworthy that the saponins mixture exhibits a weakly acidic nature (Price et al., 2009). Under alkaline conditions, acid compounds are more prone to ionization, leading to the formation of anions. These anions typically form stronger hydrations with water molecules, thereby increasing the solubility of saponins in an alkaline environment. Studies have found that many acidic saponins can be completely dissolved in a dilute alkali solution (Liu et al., 2016). On the other hand, due to the presence of hydrophobic steroid or triterpene backbone in the structure, when the pH value is adjusted to a specific level with acid, saponins will precipitate in the highly polar acidic solution. The solubility in alkaline solution and the insolubility in acidic solution therefore indicate that alkaline extraction acid precipitation may be a promising approach for the efficient separation of saponins. In fact, the method has been used to extract tea saponins from *C.oleifera* Abel cake on lab scale (Liu et al., 2016). Nevertheless, assisted strategies need to be created to improve the extraction efficiency and purity.

Three-phase partitioning (TPP) is an emerging simple, rapid, efficient and scalable technique along with an additional benefit that the solvent used can be recovered and reused (Rajendran et al., 2023). The three-phase partitioning (TPP) technique operates through sequential addition of kosmotropic salts (typically ammonium sulfate) and water-miscible organic solvents (predominantly *t*-butanol) to crude biological mixtures. This unique separation system integrates three critical downstream processing stages—fractionation, solute concentration, and primary purification—into a single unit operation. Within the TPP architecture, biomolecular constituents undergo differential partitioning based on their hydrophobicity: (1) hydrophobic metabolites accumulate in the organic solvent-enriched upper phase, (2) hydrophilic compounds concentrate in the lower aqueous phase, and (3) amphipathic proteins selectively precipitate at the liquid-liquid interface through salting-out effects and dielectric constant modification. Therefore, TPP technology combined with alkaline extraction acid precipitation may open up a new strategy for effective extraction of high-purity saponins from plant-based materials.

*Camellia oleifera* seed cake (COSC) is a oilseed presscake by-product derived from *Camellia oleifera* Abel, with an annual production of about 4 million tons worldwide (Quan et al., 2022). The COSC contains an abundant amounts of bioactive compounds, and is particularly a rich source of saponins (Quan et al., 2022). Obtaining saponin from COSC not only can offer a cost-effective natural emulsifier for the food industry but also enhances the economic value of COSC. Thus, this study proposes an integrated alkaline-three phase partitioning system combined with acid precipitation for the sustainable recovery of tea saponins from COSC. The novel approach synergizes pH-dependent solubility control with polarity-driven phase separation: alkaline conditions facilitate saponin dissolution through ionization of acidic functional groups, while the introduction of kosmotropic salts and water-miscible organic solvents establishes a triphasic system. Within this system, saponins are selectively concentrated in the aqueous phase, hydrophobic contaminants migrate to the organic phase, and amphiphilic impurities precipitate at the liquid-liquid interface. Ultrasound-assisted extraction is incorporated to enhance mass transfer efficiency during the initial dissolution stage. The aqueous phase containing enriched saponins is then subjected to acid-induced precipitation, replacing conventional acetone-based methods with an environmentally benign purification step. This integrated strategy aims to simultaneously address key challenges in saponin recovery—including high solvent consumption, toxic reagent use, and multi-step processing—while transforming COSC into value-added food-grade ingredients with emulsifying-active functionality for diverse applications.

## 2 Materials and methods

### 2.1 Materials and reagents

*Camellia oleifera* seed cake (COSC) from Jiangxi Shenzhou Tong Oil Tea Investment Corp was micronized to 425 μm particles using a rotary mill (BO-200 T) and cryopreserved at −20 °C. Analytical-grade chemicals including ammonium sulfate, *t*-butanol, and tea saponins standard (≥98 % purity) were sourced from Sinopharm Chemical and Solarbio Tech. *Escherichia coli* (ATCC#25922) provided by Guangdong Culture Collection Center.

### 2.2 Ultrasound assisted-alkaline three phase partitioning system combined with acid precipitation (UATPP-AP)

#### 2.2.1 Laboratory scale

The saponin extraction process was conducted using a multi-frequency ultrasonic reactor (25–100 kHz) integrated with alkaline three-phase partitioning through sequential parameter optimization. A precisely weighed COSC powder was mixed with pH-adjusted distilled water, followed by addition of ammonium sulfate and *t*-butanol at predetermined ratios. The mixture was homogenized and subjected to ultrasonic treatment under controlled conditions. After 1-h phase separation, the aqueous layer was acidified to precipitate saponins, which were then redissolved in alkaline deionized water and dialyzed using 500 Da MWCO membranes until achieving conductivity stabilization (<5 μS/cm) and negative sulfate ion detection via BaCl_2_ precipitation. Purified saponins were obtained by freeze-drying.

Sequential parameter optimization prioritized non-ultrasonic variables first (pH, ammonium sulfate concentration, solvent ratios, temperature, acid precipitation pH), with each optimized parameter fixed for subsequent steps (Table 1). Ultrasonic variables (frequency, energy density, duration, duty cycle) were then assessed under established non-ultrasonic conditions. NaOH concentrations were tiered by pH range (0.1–0.5 M for pH 8.0–9.0; 1.0–1.5 M for pH 10.0–11.0; 2.0 M for pH 12.0), and acoustic energy density (0.12–0.34 W/mL) was calibrated calorimetrically (Rutkowska et al., 2017).

**Table 1:** Sequential parameter optimization workflow.

Category	Parameters	Range	Optimization Order
Non-ultrasonic	pH	8.0–12.0	1
	(NH_4_)_2_SO_4_ concentration (w/v)	10 %–50 %	2
	Solute-solvent ratio (*w*/*v*)	1:2.5–1:25	3
	*t*-butanol/slurry ratio (v/v)	0.25:1–2:1	4
	Temperature (°C)	40–90	5
	Acid precipitation pH	1.0–6.0	6
Ultrasonic	Frequency (kHz)	25, 40, 60, 80, 100	7
	Acoustic energy density (W/mL)	0.12–0.34	8
	Duration (min)	10–120	9
	Duty cycle (%)	20–100	10

#### 2.2.2 Pilot scale

Pilot scale experiments were carried out in a 30 L glass extractor equipped with an agitator and combined with an ultrasound processor UIP2000hdT (Hielscher Ultrasound Technology, Germany, Teltow). The amount of extracted COSC powder was 1.5 kg, and extraction was carried out under the optimized conditions obtained from lab scale experiments. The total working volume (including COSC slurry and *t*-butanol) was approximately 22.5–25 L, utilizing 75 %–85 % capacity of the 30 L extractor to ensure safe mixing and phase separation. Specific optimized parameters are detailed in Section 3.2. The stirrer speed was 40 rpm for the effective homogenization of the mixture. After the extraction process, the mixture was allowed to stand for 1 h for separating each phase. The bottom saponins-rich phase was collected and subjected to acid precipitation. The saponins precipitate after centrifugation was desiccated in low-temperature vacuum drying and stored in a desiccator for further analysis.

#### 2.2.3 Reference extraction methods

Three reference methods i.e. alkaline three-phase partitioning and acid precipitation (ATPP-AP), ultrasound-assisted alkaline extraction and acid precipitation (UAA-AP) and conventional technique of Soxhlet extraction combined with acetone precipitation (SE-ACP) were used for comparison with UATPP-AP. Under optimized conditions (alkaline extraction at pH 10, 30 % (*w*/*v*) (NH_4_)_2_SO_4_, solute-to-solvent ratio 1:10 (w/v), *t*-butanol-to-slurry ratio 1:1 (*v*/v), 80 °C for 60 min, and acid precipitation at pH 4.5) (Fig. S1), the ATPP-AP method employed a magnetic stirrer instead of ultrasound, integrating a three-phase partitioning (TPP) system. In contrast, the UAA-AP method combined ultrasound-assisted alkaline extraction with acid precipitation, omitting the TPP system. SE-ACP extraction was carried out using 80 % ethanol as a solvent. Briefly, 5.0 g of dried COSC powder and 100 mL of the solvent were placed into a 250-mL round bottom flask fitted with a cooling condenser. The extraction was maintained at 80 °C for 6 h with gentle stirring. After the extraction process, the extract was mixed with acetone to get saponins precipitate. The pilot-scale ATPP-AP was also performed using the 30 L glass extractor as described in pilot scale UATPP-AP extraction.

### 2.3 Yield and purity determination

#### 2.3.1 Extraction yield assay

Total saponins were quantified using a modified vanillin-perchloric acid method (Hiai et al., 1976). Standard solutions (2.4 mg/mL) were serially diluted (5–25 μL) and evaporated, followed by sequential addition of 0.2 mL vanillin-acetic acid (5 %) and 1.2 mL perchloric acid. After 60 °C incubation (20 min) and ice cooling, chromophores were extracted with 5 mL ethyl acetate. Absorbance measurements at 550 nm were performed against reagent blanks using a UV–Vis spectrophotometer. The tea saponin standard curve was then obtained, and the regression equation of tea saponins standard curve was then obtained as *y* = 0.1387*x* + 0.0049 (*R*^*2*^ = 0.9988), where *y* is the concentration of tea saponins of solution for colorimetric analysis (mg/mL), and *x* is the absorbance at 550 nm. Each 20 mg sample of tea saponins extract obtained under different extraction conditions was pretreated using the same method as the tea saponins standard. Then the absorbance value (A) was obtained. The extraction yield from COSC was expressed as gram of saponins per 100 g of drying weight COSC powder (g/100 g DW) calculated using Eq. 1.

(1) $$\text{Extraction yield}\;\left(g/100g\;\mathrm{DW}\right)=\frac{C\times V}{M}\times \frac{W_{s}}{0.02}\times 100$$

where C is the total saponins content of prepared samples (g/mL), V represents the volume of deionized water used for tea saponins dissolution (mL), M is the drying weight of COSC material (g), and W_s_ refers to the weight of precipitate after precipitation, washing and drying treatment (g).

#### 2.3.2 Purity determination

Purity of the total saponins represents the saponins in the 20 mg saponins powder used in vanillin-perchloric acid method, and was calculated by the equation:

(2) $$\text{Purity}\;\left(\%\right)=\frac{V\times C}{0.02}\times 100\%$$

where V is the volume of deionized water used for saponins dissolution (mL), C represents the saponins (g/mL).

### 2.4 Solvent recovery and cyclic extraction protocol

The *t*-butanol-rich phase obtained from three-phase partitioning was acidified to pH 4.5 using 6 M HCl (1:50 *v*/v), followed by 30-min phase equilibration at 40 °C. The separated organic phase was subjected to vacuum distillation (Rotavapor® R-300, 82.5 °C, 0.09 MPa) with 3 Å molecular sieves (1:10 *w*/*v*, 2 h dehydration) to obtain regenerated solvent. Five consecutive extraction cycles were conducted using recycled *t*-butanol under optimized UATPP-AP conditions. Saponin yield and purity as well as interfacial tension and in vitro antioxidant activity were then determined.

### 2.5 Structural characterization

#### 2.5.1 Fourier transform infrared spectroscopy (FTIR)

Saponin samples were homogenized with KBr at 1 % (*w*/w) ratio and pressed into transparent pellets. Spectra acquisition was performed on a Bruker Tensor 27 spectrometer with the following parameters: 32 scans per measurement, spectral range of 4000–400 cm^−1^, and resolution of 2 cm^−1^. Triplicate measurements were conducted for each specimen to ensure reproducibility.

#### 2.5.2 High-performance liquid chromatography (HPLC)

The chromatographic fingerprint was obtained using an Agilent 1200 HPLC system. Pretreatment involved dissolving 50 mg saponins sample in 5 mL 70 % methanol (*v*/v), followed by filtration through 0.22 μm PTFE membranes. Separations were achieved on a ZORBAX SB-C18 column (250 × 4.6 mm, 5 μm) with acetonitrile/water gradient elution (20 %–100 % acetonitrile in 40 min) at 1.0 mL/min (30 °C). UV detection at 215 nm enabled structural identification through retention time alignment with certified reference standard.

### 2.6 Interfacial tension measurement

Interfacial properties were characterized using a DCAT21 tensiometer (Data Physics, Germany) with platinum Wilhelmy plate geometry (9.95 × 0.2 mm). Test systems comprised 10 % peanut oil versus aqueous surfactant solutions (0.0005 %–5 % *w*/w). Following ISO-compliant cleaning protocols, the oil phase was carefully layered onto surfactant-containing aqueous phase until complete plate immersion. Detachment force measurements at 25.0 ± 0.1 °C were converted to interfacial tension values through instrument-integrated Laplace equation calculations.

### 2.7 Emulsifying properties measurements

Water-in-oil emulsions were formulated through sequential high-shear mixing (900 rpm × 5 min) and ultrasonication (30 kHz, 250 W, 70 % amplitude × 10 min) of 10 % peanut oil with surfactant solutions (0.5–5 wt% saponin extracts or 0.2–2.5 wt% Tween 80). Freshly prepared emulsions underwent 14-day ambient aging (20 ± 1 °C) for macroscopic stability evaluation. Particle size characterization was performed via laser diffraction (Beckman Coulter LS 13320) with Sauter mean diameter (d_32_) calculations according to established protocols (Salminen et al., 2020).

### 2.8 Assay of in vitro antioxidant activity

Antioxidant capacity was comprehensively assessed through three validated methodologies. DPPH (1,1-diphenyl-2-picrylhydrazyl) radical scavenging activity was quantified via absorbance measurements at 517 nm (Brand-Williams et al., 1995), with scavenging efficiency calculated as:

(3) $$I\%=\left[1-\frac{A_{i}-A_{j}}{A_{c}}\right]\times 100\%$$

where A_i_, A_j_ and A_c_ represent the absorbance of control, sample with DPPH, and sample blank, respectively. Dose-response curves (IC_50_) were established using vitamin C as positive controls. TEAC (Trolox equivalent antioxidant capacity) values were determined through 2,2′-Azino-bis(3-ethylbenzothiazoline-6-sulfonic acid) (ABTS^+^) decolorization kinetics (Re et al., 1999), expressed as mg Trolox equivalents/g sample. Concurrently, FRAP (Ferric reducing antioxidant power) analysis (Benzie & Strain, 1996) quantified ferric ion reduction capacity, with results standardized as μmol Fe^2+^/g sample using Trolox calibration curves. All measurements were normalized to 100 g dry weight basis for comparative analysis.

### 2.9 Antibacterial assay

Extracts were assayed for antibacterial activity using the agar well diffusion technique against *E.coli*. In brief, a stock solution of 10 g/L concentration of each extract was prepared, from which varying concentrations were achieved. The UATPPS sample (obtained by UATPP-AP method) contained UATPPS at concentrations of 0.1–5.0 g/L, giving a range of total saponins concentrations from 0.084 to 4.39 g/L. The tea saponins standard was prepared with a concentration of 3.0 g/L, whereas the concentrations of the extracts TPPS (obtained by ATPP-AP method), UAA-APS (obtained by UAA-AP method) and SES (obtained by conventional SE-ACP method) were set at 5.0 g/L. For antibacterial activity assay, sterile oxford cups were placed on the LB plates that had been pre-inoculated with *E. coli* (inoculated density of OD_600_ was approximately 0.08), and then each cup was added with 200 μL samples. Sterile distilled water was taken as the control. All plates underwent 12 h incubation at 37 °C, with zone diameters measured using digital calipers. Each test sample was assayed in quadruplicate.

### 2.10 Statistical analysis

All experiments were carried out in three replicates unless otherwise stated and the mean ± standard deviation was used in the analysis. Significant differences between groups were assessed through one-way ANOVA with Tukey's post-hoc test in OriginPro 9.0 (OriginLab). Statistical significance thresholds were established at α = 0.05 confidence level, with *P*-values<0.05 considered statistically significant differences.

## 3 Results and discussion

### 3.1 Laboratory scale UATPP-AP extraction

The factors that affect the extraction yiled and purity of saponins from COSC were divided into nonultrasonic factors and ultrasonic factors. In the study, the nonultrasonic factors involved alkali extraction pH, ammonium sulfate concentration, solute to solvent ratio, *t*-butanol to slurry ratio, temperature and acid isolation pH. The ultrasonic factors included ultrasonic frequency, acoustic energy density (AED), ultrasound duration (time), and treatment mode (duty cycle).

#### 3.1.1 Optimization of the nonultrasonic factors

**Fig. 1:**
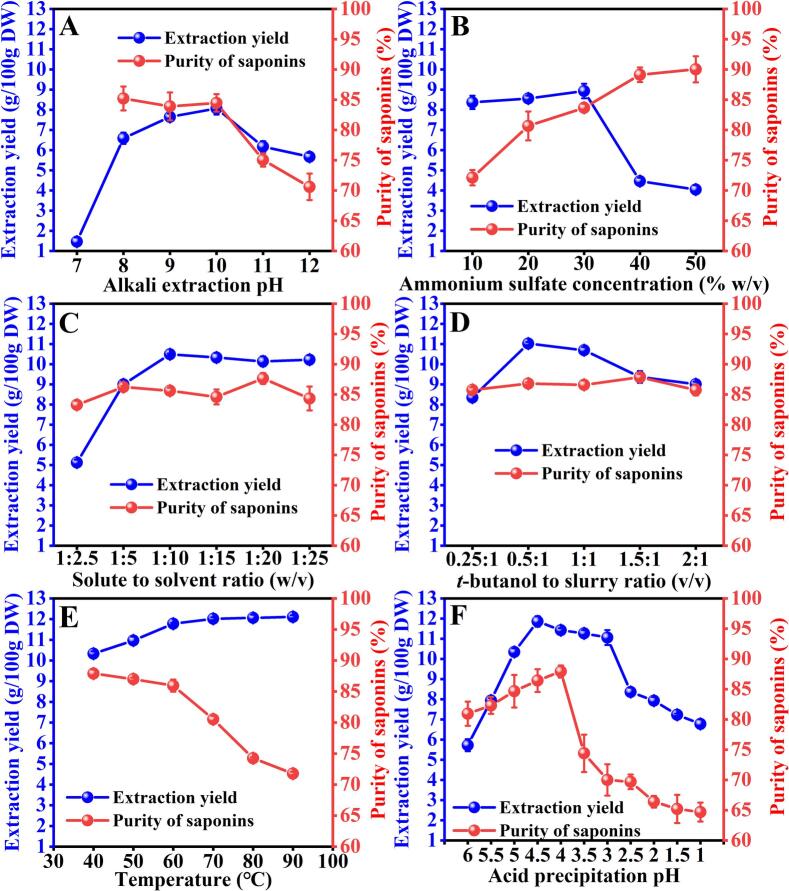
Effects of nonultrasonic factors including alkali extraction pH value (A), (NH_4_)_2_SO_4_ concentration (%, *w*/*v*) (B), solute to solvent ratio (*v*/v) (C), *t*-butanol to slurry ratio (v/v) (D), temperature (E) and acid precipitation pH (F) on the extraction yield (g/100 g dry weight of COSC powder) and purity (%) of saponins extracted from *Camellia oleifera* seed cake (COSC).

The effect of alkali extraction pH value on saponins extraction was presented in Fig. 1A. The solvent with deionized water (pH 7.0) was able to extract only 1.46 ± 0.21 g of saponins per 100 g of dry weight, confirming the relatively low solubility of tea saponins in neutral warm (50 °C) water due to their amphiphilic molecular structure (Timilsena et al., 2023). The saponins extraction yield increased from 6.59 ± 0.28 % to 8.07 ± 0.31 % g/100 g DW with increasing pH values from 8 to 10, suggesting that increasing the concentration of alkaline media could improve the extractability of saponins from COSC. However, further pH increasing resulted in yield rapid decrease. These results implied that a low pH value below 8 decreases the solubility of tea saponins, thereby lowering the extraction yield. Conversely, the observed yield reduction at pH >10 aligns with reported alkaline hydrolysis of triterpenoid glycosides, where high pH values promote structural degradation (Liu et al., 2016). Fig. 1A also shows the effect of alkali solution pH value on the purity of tea saponins. It can be seen that the purity of the saponins changed insignificantly with the increase of alkali solution pH from 8 to 10, after that it decreased significantly (*P* < 0.05). The decrease was likely due to the degradation of tea saponins under strong alkaline conditions (Savarino et al., 2023). Considering the yield and purity of tea saponins, the appropriate alkali solution pH value should be around 10.

The effect of varying concentration of (NH_4_)_2_SO_4_ (%, *w*/*v*) on the extraction yield and purity was shown in Fig. 1B. The extraction yield did not change significantly when (NH_4_)_2_SO_4_ was varied from 10 % to 30 %. In TPP system, addition of salt with high concentration to the aqueous mixture is believed to be able to increase the solid liquid mass transfer, since it can gives an osmotic shock to the cells leading to bursting or rupture of cells (Panadare & Rathod, 2017). The result here can be interpreted as that mechanical, cavitation, and thermal effects induced by ultrasound are so strong in enhancing mass transfer across cell membranes that the osmotic shocks (NH_4_)_2_SO_4_ generated can be neglected. When the (NH_4_)_2_SO_4_ concentration exceeded 30 %, the extraction yield declined rapidly. The decrease can be attributed to the pronounced kosmotropic effect resulting from high concentration of (NH_4_)_2_SO_4_, which leads to an increased binding of sulfate ions with hydrogen bonds of water molecules and consequently reducing the availability of water molecules for dissolving saponins (Rajendran et al., 2023). The increase of the (NH_4_)_2_SO_4_ concentration in the system improved the purity (Fig. 1B). The reason for getting higher purity of saponins in high concentration is that, with the increase in (NH_4_)_2_SO_4_ concentration, the “salting out” effect was strengthened. And this will help to transfer macromolecular proteins, oil, weakly polar compounds, etc., out of the lower aqueous phase, thus making the purity of the saponins enhanced (Panadare & Rathod, 2017). Although the maximum saponins purity (90.02 ± 2.16 %) was achieved at 50 % concentration, the extraction yield (4.05 ± 0.21 g/100 g DW) was much lower than that at 30 % (8.93 ± 0.36 g/100 g DW). Considering that saponins purity at 30 % concentration was 84.66 ± 0.68 %, only around 6 % lower than that at 50 %, 30 % (*w*/*v*) (NH_4_)_2_SO_4_ was selected as the optimal concentration.

The variation of solute to solvent ratio i.e. amount of COSC powder to NaOH solution on the extraction yield and purity of saponins by UATPP-AP was shown in Fig. 1C. Saponin purity remained stable across tested solvent volumes, while yield demonstrated solvent-dependent behavior with a characteristic peak at 1:10 solute-to-solvent ratio. Beyond this critical ratio, yield showed marginal decline (9.2 % reduction, *P* > 0.05) despite continued solvent addition, indicating optimal extraction efficiency at the identified ratio threshold. Theoretically, for a fixed amount of solid matrix, the more amount of solvent used, the more dilute effect in the solvent side (Lou et al., 2009). This created a larger concentration difference between the interior of the solid and the external solvent, and that increases the driving force for the mass transfer. Thus a faster extraction yield could be obtained at a comparatively higher quantity of solvent. However, if the solution was very dilute, an extra solvent increase would not result in a sufficient increase in the concentration difference, thus the increase in mass transfer would be limited (Cacace & Mazza, 2003). Therefore, at solute to solvent ratio above than 1:10, no major influence on the extraction yields was obtained. Consequently, 1:10 (*w*/*v*) was regarded as the optimum solute to solvent ratio for the subsequent experiments.

Effect of *t*-butanol on extraction yield and purity was studied by varying ratio of *t*-butanol to slurry as 0.25:1, 0.5:1, 1:1, 1.5:1 and 2:1 (*v*/v). As depicted in Fig. 1D, the extraction yield was highest at 0.5:1 ratio, and with further increase in the proportion of *t*-butanol, it decreased significantly (*P* < 0.05). This aligns with the established polarity window for optimal three-phase partitioning (TPP), where intermediate *t*-butanol levels balance phase separation efficiency and compound solubility (Rajendran et al., 2023). Mechanistically, suboptimal *t*-butanol concentrations (<0.5:1) compromise ammonium sulfate's salt-out capacity by weakening cooperative hydration effects, whereas excess *t*-butanol (>0.5:1) induces over-structuring of interfacial water layers through enhanced kosmotropicity, thereby restricting polar compound diffusion. Moreover, within the ultrasound assisted TPP system, an excess of *t*-butanol can result in elevated surface tension (Yan et al., 2017), which is detrimental to the formation of high-intensity ultrasonic cavitation and consequently leads to a reduced extraction yield. Notably, saponin purity remained constant (82 %–84 %) across all tested ratios (Fig. 1D), confirming *t*-butanol's primary role in phase partitioning rather than selective purification. These findings collectively justify the 0.5:1 ratio selection for subsequent experiments.

Temperature plays a vital role for saponins leaching. The temperature of the UATPP-AP method was selected in the range between 40 °C and 90 °C. As shown in Fig. 1E, when the temperature was low (40 °C–70 °C), the extraction yield significantly increased (*P* < 0.05) with temperature increasing; further increases in the temperature, however, led to no significant improvement (*P* > 0.05). The purity of saponins varied only slightly at temperatures<60 °C; however, as the temperature continued to rise, it declined sharply. This can be attributed to the dissolution of impurities under elevated temperatures. A similar observation was observed in previous study, where high temperatures cause partial precipitates in the middle layer to dissolve again in the lower phase (Gul et al., 2022). Considering the yield and purity, a temperature of 60 °C was defined as the optimal condition.

To determine the optimal pH value for saponins precipitation post-extraction, a systematic evaluation of eleven pH values ranging from 1.0 to 6.0 was conducted. As shown in Fig. 1F, the tea saponins had minimum solubility at around pH 4.5, which resulted in 11.86 ± 0.24 g/100 g DW recovery. Saponins are weak acidic substances characterized by a hydrophobic steroid or triterpene backbone. This structural feature results in a marked decrease in its solubility in aqueous solutions when the pH decreases into the acidic range. However, it is not the case that the lower the pH, the better. Saponins undergo partial hydrolysis or resolubilization in solutions of high acidity (Aziz et al., 2019). Saponins purity tends to increase as the pH value decreases from 6 to 4, after that it decreased significantly. The pronounced reduction in the purity at pH 3 and 3.5 may be attributed to the co-precipitation of other impurities, as the extraction efficiency did not exhibit a significant decline within this pH range. The decrease in purity at pH levels below 3.0 could potentially result from hydrolysis or re-dissolution of tea saponins. Taken together, acid precipitation pH value of 4.5 was considered optimal.

#### 3.1.2 Optimization of the ultrasonic factors

**Fig. 2:**
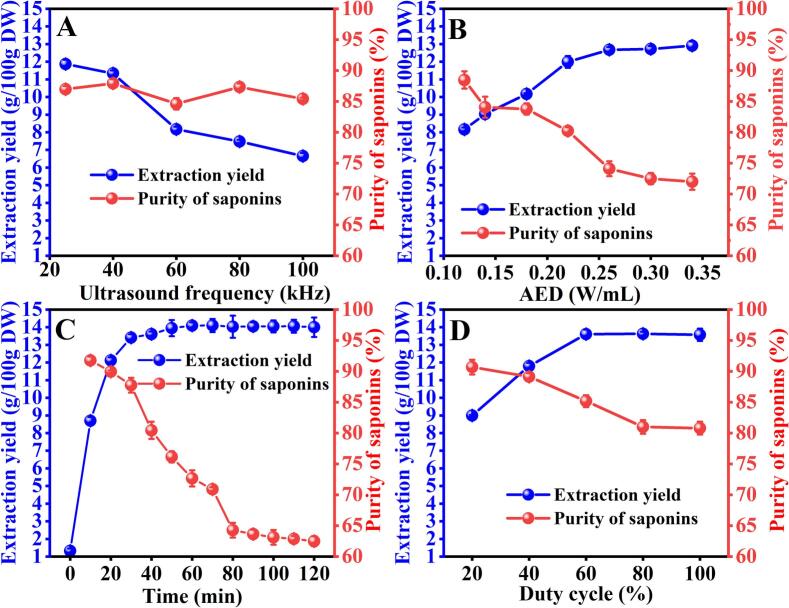
Effects of ultrasonic factors including ultrasound frequency (A), acoustic energy density (AED) (B), ultrasound duration (C) and duty cycle (D) on the extraction yield (g/100 g dry weight of COSC powder) and purity (%, *w*/w) of saponins extracted from *Camellia oleifera* seed cake (COSC).

Fig. 2A shows the effects of ultrasound frequency (25, 40, 60, 80, and 100 kHz) on the extraction yield and purity when other parameters were kept constant. It can be found that the purity at different frequencies did not change significantly (*P* > 0.05). Nevertheless, ultrasonic frequency significantly influences the extraction yield. As the ultrasonic frequency increased from 25 to 40 kHz, the yield of saponins slightly decreased (*P* > 0.05); however, as the frequency was further increased, the yield dropped rapidly (*P* *<* 0.05). The result shows that lower ultrasonic frequencies are conducive to the saponins extraction. Previous studies have reported similar trend in their research where low ultrasound frequency was found to be preferable (Delran et al., 2023; Liao et al., 2022). The reason behind this can be that the high ultrasound frequency makes the length of compression rarefaction phase (during which cavitation bubbles grow) too short to obtain the required size of the microbubbles, thus causing the formation of less violent cavitation bubbles (Medina-Torres et al., 2017). Moreover, high ultrasonic frequencies increase solvent vapor pressure, filling cavitation bubbles with vapor instead of vacuum. This reduces bubble collapse intensity, lowering extraction efficiency. Therefore, 25 kHz was chosen as the optimal frequency for subsequent experiments.

Acoustic energy density (AED) was used to express the acoustic energy dissipated in the extraction system. Effects of AED at different levels on the extraction yields are plotted in Fig. 2B. When AED was raised from 0.12 to 0.26 W/mL, the recovery of total saponins displayed steady increase from 8.16 ± 0.11 to 12.68 ± 0.25 g/100 g DW. Apparently, AED was critical in improving the saponins yield. The intensification of extraction efficacy can be attributed to the larger amplitude ultrasound waves generated at high AED. When larger waves propagate in the solvent, a more pronounced cavitation effect will ensues, leading to a high solid-liquid mass transfer ratio and thereby facilitating the dissolution of more target compounds into the solvent (Mehta et al., 2022). Nevertheless, after 0.26 W/mL of ultrasonic energy, the increase in extraction yield was insignificant (*P* > 0.05), which can be owing to the limitations of equilibrium extraction. Contrary to the extraction yield, the purity declined with AED increasing, and varied little after it surpassed 0.30 W/mL. The result indicated that increase in ultrasonic energy was unfavourable for the purity of saponins in the aqueous phase. This could be attributed to the fact that as the ultrasonic energy increases, the enhanced solid-liquid mass transfer also expedites the dissolution process of other polar compounds in the aqueous phase. Based on these findings, the acoustic energy density of 0.22 W/mL was chosen as the optimal parameter.

The effects of ultrasound duration (time) on the extraction yield and purity were illustrated in Fig. 2C. The extraction yield sharply increased during the first 20 min, nearly approaching the maximum concentration; then it increased slowly until reaching the maximum at 60 min. The initial extraction surge primarily resulted from immediate solubilization of surface-available and cell wall-disrupted saponins, whereas subsequent yield stabilization reflected intracellular mass transfer limitations through undamaged cellular membranes in *C.oleifera* cells. Beyond 60 min, no significant change was noticed. As the ultrasound duration continued to increase, the purity showed decrease (Fig. 2C). We observed that almost 85 % of the total saponins was obtained within 20 min, and when ultrasound duration increasing from 20 to 30 min, there was still a considerable increase from 12.12 ± 0.17 to 13.41 ± 0.19 g/100 g DW; Nevertheless, when ultrasound duration was reached at 40 and 50 min, the total yields were only marginally higher than that at 30 min, while the purity at these two ultrasound durations (80.31 ± 1.45 % and 76.15 ± 0.92 % respectively) were significantly lower than that at 30 min (87.76 ± 1.23 %). Therefore, an ultrasound duration of 30 min was determined to be optimal.

Ultrasonication on the continuous mode directs to an increase in temperature which might degrade saponins. Therefore, ultrasonication on the pulse mode was chosen. Evaluation of duty cycle parameters (20 %–100 %) demonstrated two operational phases: tea saponin yield increased significantly (*P* < 0.05) up to 60 % duty cycle (6 s ON/4 s OFF), followed by a yield plateau (*P* > 0.05) at higher values. This stabilization suggests complete dissolution of extractable saponins at 60 % duty cycle, with no additional yield improvement despite increased energy input. Purity decreased gradually from 90.68 ± 1.21 % (20 % duty cycle) to 85.17 ± 0.95 % (60 %), stabilizing at 83 %–84 % beyond 80 % duty cycle. The 60 % duty cycle was selected as optimal, balancing peak yield (13.51 ± 0.19 g/100 g DW) with acceptable purity retention (85.17 ± 0.95 %).

### 3.2 Comparison with reference methods in extraction yield and purity

**Fig. 3:**
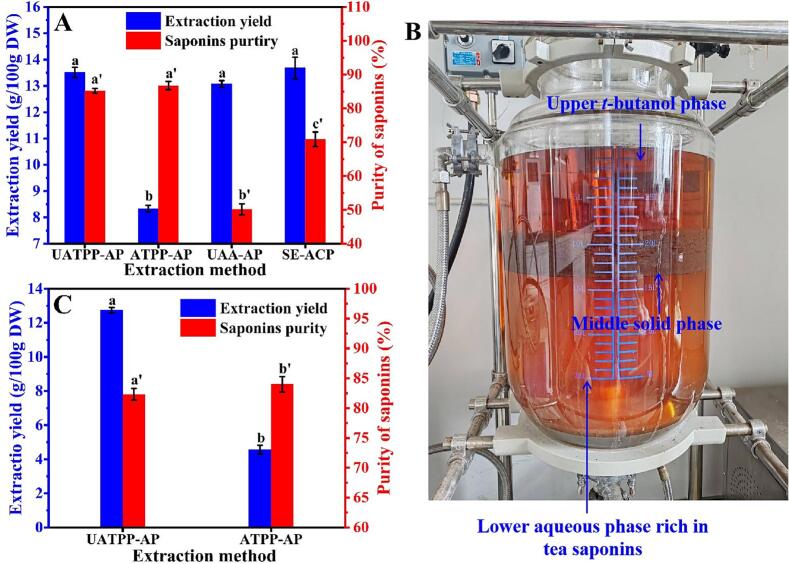
Comparison of UATPP-AP with other methods in extraction yield and purity at lab scale (A), three phases formed after pilot scale UATPP-AP extraction (B), and extraction yields and purity of saponins obtained in pilot scale (C). Different lowercase letters (e.g., a, b) for extraction yield and lowercase letters with primes (e.g., a′, b′) for saponin purity indicate statistically significant differences within the same group (*P* < 0.05).

The maximum extraction yield of saponins was achieved under the conditions of alkali extraction pH 10, (NH_4_)_2_SO_4_ concentration 30 % (*w*/*v*), solute to solvent ratio 1:10 (w/v), *t*-butanol to slurry ratio 0.5:1 (*v*/v), temperature 60 °C, acid precipitation pH 4.5, ultrasound frequency 25 kHz, acoustic energy density 0.22 W/mL, duration 30 min and duty cycle 60 %. Under these conditions, the lab-scale saponins extracted from the *C. oleifera* seed cake was 13.51 ± 0.19 g/100 g DW, with a purity of 85.17 ± 0.95 % (Fig. 3A). For comparison with the UATPP-AP approach, ATPP-AP, UAA-AP and classical technique of Soxhlet extraction combined with acetone precipitation (SE-ACP) were conducted. ATPP-AP was performed in an analogous way with UATPP-AP, where agitation was utilized instead of ultrasound. As represented in Fig. 3A, ATPP-AP at optimized conditions yielded saponins 8.33 ± 0.12 g/100 g DW (Fig. S1), which was significantly lower than that of UATPP-AP. Moreover, ATPP-AP required nearly 60 min for getting maximum extraction and it also required a higher extraction temperature (80 °C, Fig. S1E). UAA-AP and UATPP-AP had a similar extraction yield, indicating that the application of TPP did not alter the terminal extraction yield. However, UAA-AP gave a lower purtiy (50.13 ± 1.62 %) than that of the UATPP-AP (Fig. 3A). This result demonstrated that the combination of TPP system in alkaline extraction and acid precipitation method can help improve the purity of tea saponins. Conventional SE-ACP could be the most widely used method for saponins purification (Yu & He, 2018a). SE-ACP process resulted in tea saponins 13.68 ± 0.41 g/100 g DW, with purity of 76.87 %. Although the SE-ACP process yielded a comparable amount of saponins, it required a significantly higher temperature (80 °C) and a substantially longer extraction time (6 h) compared to UATPP-AP, which operated at 60 °C for only 30 min. Compared with previously reported studies, the current UATPP-AP also demonstrates superior performance. For instance, conventional ethanol-water extraction requires higher operational temperatures (82.2 °C) and longer processing times (3.3 h) (Hu et al., 2012), while deep eutectic solvent extraction achieves a yield of 9.4 g/100 g and with a purity below 80 % (Yu et al., 2023).

Taken together, the integration of ultrasound can result in a significant reduction in extraction time, while the incorporation of the TPP system can enhance the purity of saponins. It is well known that ultrasound favors extraction by enhancing mass transfer during the solid-liquid extraction process (Korobiichuk et al., 2023). For TPP system, it provides a new medium with hydrophobicity increasing gradually from bottom to top three phases, allowing much high selectivity for solutes (Yan et al., 2017). So, the increase in the purity of tea saponin may be attributed to the transfer of impurities such as proteins, polypeptides and amino acids from the aqueous phase to the middle precipitate phase, resulting in less impurities co-precipitating with the tea saponins when the pH drops to 4.5.

### 3.3 Scale-up of the optimized system

To demonstrate pilot scale feasibility of the new process, a scale-up experiment was carried out under the optimized conditions. In this sense, 1.5 kg dried COSC sample was mixed with 15 L of water (pH = 10) in an ultrasound-assisted extraction vessel. After the extraction process, three distinct phases were formed: a upper *t*-butanol phase, a middle solid phase and a lower aqueous phase. Both the upper and lower phases exhibited a reddish-brown hue, with the lower phase displaying a slightly lighter shade (Fig. 3B). Saponins were mainly distributed in the lower aqueous phase. As presented in Fig. 3C, the extraction yield and purity obtained from the pilot-scale were 12.74 ± 0.16 g/100 g DW and 82.31 ± 0.98 %, respectively, which are comparable to the lab-scale values. The minimal yield deviation (5.6 % reduction from lab-scale 13.5 g/100 g) suggests robust preservation of cavitation effects and phase separation dynamics in large-volume systems, likely due to ultrasound-enhanced interfacial turbulence compensating for scaling-related viscosity gradients (Zhang et al., 2022). This result indicated that the upscaling of the newly established method for saponins extraction from laboratory to pilot scale was feasible.

Parallel ATPP-AP trials at pilot scale were conducted for comparative analysis. The saponin yield obtained through pilot-scale ATPP-AP (4.56 ± 0.25 g/100 g DW) was substantially lower than its laboratory-scale counterpart (8.33 ± 0.12 g/100 g DW). Under identical operational parameters except for ultrasound application, the amplified performance differential between scaled systems, quantified as a 179 % yield improvement through ultrasound assistance at pilot scale (UATPP-AP: 12.74 g/100 g DW vs. ATPP-AP: 4.56 g/100 g DW) compared to a 64 % enhancement observed at laboratory scale (UATPP-AP: 13.5 g/100 g DW vs. ATPP-AP: 8.33 g/100 g DW), conclusively demonstrates ultrasound's capability to disrupt boundary layer formation in viscous media. This mechanism is particularly critical in large-scale systems where conventional agitation fails to maintain adequate shear rates (Humphrey, 2007). This clearly showed that ultrasound technology significantly enhanced the extraction efficiency of saponins from *C. oleifera* seed cake in a large-scale ATPP-AP system.

Taken together, UATPP-AP has the merits of easy to scale up, high extraction ability, short extraction time, and low extraction temperature, which can be an attractive option for tea saponins recovery from the food by-product COSC. Notably, the 2-h processing cycle achieves an 80 % time reduction compared to commercial ethanol extraction methods while maintaining >82 % purity without chromatographic purification, representing a breakthrough in industrial-scale biosurfactant production. The technology's 60 °C operational temperature minimizes thermal degradation risks relative to conventional saponin extraction processes that typically exceed 80 °C (Chi et al., 2023). Future investigations should prioritize energy-efficient cavitation management strategies for reactor volumes exceeding 100 L, particularly addressing ultrasound signal attenuation mechanisms in high-viscosity media.

### 3.4 Solvent recovery and reusability in saponins extraction

The *t*-butanol recovery was implemented through established acid-mediated phase separation (pH 4.5) followed by conventional vacuum distillation (82.5 °C), achieving 91.2 ± 2.1 % solvent recovery rate with pharmaceutical-grade purity (>98 %) (Table 2). Recycled solvent maintained saponin extraction efficiency at 12.56 ± 0.49 g/100 g DW through five successive batches, while preserving functional performance: interfacial tension (5.89 ± 0.81 mN/m) and antioxidant capacity (IC_50_ 1.28 g/L) showed no significant difference from fresh solvent controls (*P* > 0.05). Compared with traditional extraction solvents (acetone/n-hexane) that are notoriously difficult to recycle (Aziz et al., 2019), the UATPP-AP method thus demonstrates a viable approach for achieving solvent residue-free green manufacturing of functional ingredients in the food industry.

**Table 2:** Key parameters of *t*-butanol recycling and functional performance in saponins extraction.

Parameter	*t*-Butanol (Recycled)	Fresh *t*-Butanol (Control)	Acetone/n-Hexane (Traditional)⁎
Recovery efficiency (%)	91.2 ± 2.1		<50
Saponins yield (g/100 g DW)	12.56 ± 0.49 (Cycle 5)	12.74 ± 0.16	Approximately 10.0
Saponin purity (%)	77.37 ± 0.71	>80	75–85
Interfacial tension (mN/m)	5.89 ± 0.81	5.21 ± 0.43	>7.0
Antioxidant IC50 (g/L)	1.38	1.23	>1.5

⁎ Data from Aziz et al. (2019).

### 3.5 Characterization of the extracted saponins

#### 3.5.1 FTIR analysis

**Fig. 4:**
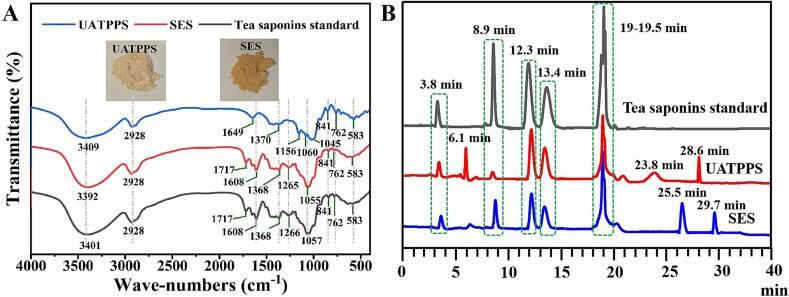
FTIR absorption spectra (A) and HPLC analysis of the extracted saponins (B). UATPPS: saponins extract obtained using ultrasound-alkaline three-phase partitioning coupled with acid precipitation (UATPP-AP); SES: saponins extract obtained using conventional soxhlet extraction combined with acetone precipitation (SE-ACP).

Tea saponins extracted from UATPP-AP and conventional SE-ACP process, designated UATPPS and SES, were subjected for FTIR analysis. FTIR spectrograms were illustrated in Fig.4A. Curves of UATPPS and tea saponins standard were consistent in trend with local differences. UATPPS had infrared absorption peaks at 3409, 2928, 1649, 1370, 1156, 1060, 1045, 841,762 and 583 cm^−1^. Among them, the broad peak near 3400 cm^−1^ corresponded to a hydroxyl stretching vibration, while peak of 2928 cm^−1^ was assigned to a methyl or methylene stretching vibration. In the wave-numbers range from 2500 cm^−1^ to 1900 cm^−1^, no characteristic absorptions were observed, suggesting the absence of triple or double bond accumulations. The unique peak at 1649 cm^−1^ is characteristic of C=C. Despite the absence of a peak at 1649 cm^−1^ in both the tea saponins standard and SES, previous studies have reported an infrared absorption peak for tea saponins at around 1649 cm^−1^ (Li et al., 2021; Yu & He, 2018b). Both SES and tea saponins standard exhibited C=O characteristic peaks at 1717 cm^−1^ and 1608 cm^−1^. No peak indicative of C=O stretching vibration was observed in UATPPS. This absence may account for the lighter color of UATPPS compared to SES (Fig. 4A), as the lack of carbonyl groups could influence the conjugation effect. The characteristic absorption peak at 1370 cm^−1^ was owed to the antisymmetric bending vibrations of the methyl group (-CH_3_). The absorption bands observed between 1000 and 1200 cm^−1^ corresponded to C-O-C stretching vibrations characteristic of primary alcohols (Li et al., 2021), confirming the glycosidic linkages in sapogenin structures. UATPPS also displayed peak at 841 cm^−1^, which was attributed to the β-glycosides. These findings align with previously published spectra of oleanane-type tea saponins (Yu et al., 2023; Yu & He, 2018b).

#### 3.5.2 HPLC analysis

HPLC analysis was also employed to confirmed that the UATPPS represents oleanane-type triterpenoid saponin mixtures, validated through chromatographic alignment with commercial oleanane-type triterpenoid saponins standard (Fig. 4B). The standard exhibited characteristic peaks corresponding to a polar aglycone derivative (3.8 min, 4.5 %), monodesmosidic camelliasaponin B1 (8.9 min, 21.6 %), bidesmosidic theasaponins E2 (12.3 min, 16.5 %) and E1 (13.4 min, 10.8 %), along with acetylated derivatives (19–19.5 min, 45.1 %), collectively accounting for 98.5 % total peak area. While UATPPS retained all key saponin markers, it showed an additional prominent peak at 6.1 min and minor peaks at 23.8/28.6 min. Mechanistically, the 6.1 min peak reflects TPP-mediated preservation of hydrophilic saponins typically lost during acetone precipitation due to solubility hysteresis, whereas the late minor peaks may arise from ultrasound-induced acyl migration (Bera et al., 2015). In contrast, SE-ACP-derived SES displayed unique peaks at 25.5/29.7 min, likely representing oxidation-aggregated saponin-polyphenol/protein complexes formed during acetone treatment. Notably, the elevated peak intensities of E1 and E2 observed in UATPPS relative to SES (Fig. 4B) indicate that the UATPP-AP method effectively enhances the retention of these critical bidesmosidic saponins, which are associated with improved emulsifying properties (Kanlayavattanakul et al., 2024) and antioxidant capacity (Wang et al., 2024).

#### 3.5.3 Interfacial properties

**Fig. 5:**
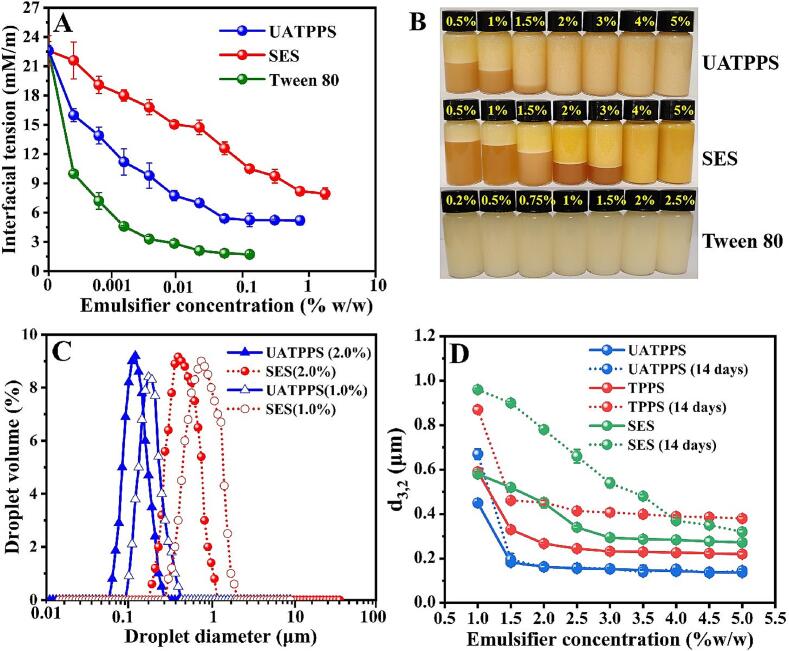
Interfacial and emulsifying properties of UATPPS. (A) Interfacial tension as a function of concentrations measured at the oil-water interface; (B) Physical stability of emulsions after 14 days of storage; (C) Comparison of particle size distribution in 10 wt% peanut oil-in-water emulsions stabilized by UATPPS and SES at concentrations of 2 wt% and 1 wt%; (D) Mean particle size (d_3,2_) of 10 wt% peanut oil-in-water emulsions stabilized by different concentrations of the extracted saponins.

The interfacial characteristics are critical in determining the ability of a surfactant to fabricate and stabilize emulsions (Cui et al., 2023). Therefore, the interfacial behavior of the saponins extract (UATPPS) was systematically investigated through comparative analysis with SES obtained by the traditional method SE-ACP and synthetic Tween 80. As shown in Fig. 5A, in the absence of surfactant, the interfacial tension was 22.58 ± 0.02 mN/m. The interfacial tension decreased steeply as the concentraction of UATPPS was first increased. In emulsion systems, interfacial tension of an emulsifier indicates whether it is easy to form an emulsion, and an ideal emulsifier is capable of rapidly adsorbing to the interface and reducing the interfacial tension (Cui et al., 2023). The sharp decline of interfacial tension therefore demonstrated that UATPPS has outstanding ability to form stabilize emulsion. With further increase in the concentration, a relatively constant value was attained, indicating that surfactant accumulation at the oil-water interface approached adsorption saturation capacity. In case of SES, interfacial tension declined as the concentration increased, but the values of this decline was less more obvious. The critical micelle concentration (CMC), as determined from the plateau region of the interfacial tension-concentration curves (Fig. 5A), was approximately 1.0 wt% for UATPPS and approximately 3.0 wt% for SES. This lower CMC value suggests enhanced interfacial adsorption efficiency. The CMC value observed in this study remained comparable to that reported in the pioneering work by Zhang et al. (2025), where tea saponins extracted from COSC using ultrasonic-assisted enzymes exhibited a CMC of 0.5 wt%, highlighting the competitive performance of the extraction method UATPP-AP. For synthetic Tween 80 (control group), the CMC was below 0.5 % *w*/w, which is consistent with its superior equilibrium interfacial tension (1.26 ± 0.07 mN/m). These variations in CMC directly influence the required dosage of emulsifiers in practical applications (see Section 3.5.4).

#### 3.5.4 Emulsion formation and storage stability

The emulsifying capacity and emulsion stability of UATPPS were compared with SES and Tween 80. The emulsifying capacity was investigated in oil-in-water emulsions (10 % w/w peanut oil and pH 7) by varying concentrations at 0.5 %–5.0 % (*w*/w) for saponins extracts and at 0.2 %–2.5 % (w/w) for Tween 80. Initially, uniform emulsions can be successfully formed at all concentrations of the three emulsifiers. Emulsions from UATPPS and SES were dark brown, while the Tween 80 emulsion was white. After storage for 14 days, the emulsion containing Tween 80 maintained stability at low concentrations, demonstrating superior emulsifying properties (Fig. 5B).

CMC and hydrophilic-lipophilic balance (HLB) are pivotal parameters governing emulsion performance (Shi et al., 2019). In the case of saponins extracts, phase separation was observed in the lower layer of emulsions containing <1.5 % (w/w) UATPPS and < 4.0 % (w/w) SES (Fig. 5B). This threshold concentration difference directly aligns with their CMC values (approximately 1.0 % for UATPPS vs. approximately 3.0 % for SES, Section 3.5.2), where UATPPS achieves interfacial saturation near its CMC, while SES requires concentrations exceeding its higher CMC. The lower CMC of UATPPS reflects its enhanced surfactant efficiency, enabled by higher saponin purity (Fig. 3A). The HLB-driven stabilization mechanism further elucidates these differences. Saponins naturally possess a balanced HLB through their amphiphilic structure-hydrophobic triterpene aglycones anchor at oil phases, while hydrophilic oligosaccharide chains stabilize water phases (Akbari et al., 2023). In UATPPS, elevated purity optimizes this inherent HLB by maximizing functional saponin availability, whereas SES's lower purity disrupts HLB efficiency through impurity-mediated competitive adsorption.

The enhanced emulsifying activity of UATPPS was further substantiated through particle diameter measurements. Specifically, at equivalent surfactant concentrations of 2.0 wt% or 1.0 wt%, the droplet sizes of UATPPS-stabilized emulsions were significantly smaller than those of SES-stabilized emulsions (Fig. 5C). Notably, UATPPS concentrations ranging from 1.5 % to 5.0 % yielded small oil-in-water emulsion droplets with a mean diameter (d_3,2_) below 0.20 μm (Fig. 5D). These dimensions surpass those of conventional saponin-based emulsifiers, as evidenced by previous studies reporting d_3,2_ values exceeding 0.20 μm for tea saponin-stabilized and quillaja saponin-stabilized emulsions under comparable storage conditions (Zhu et al., 2018). In contrast, TPPS (ATPP-AP extract, 84.7 % purity) at 2.0–5.0 % concentrations produced droplets within 0.22–0.28 μm initially, but exhibited progressive coalescence during storage (d_3,2_ = 0.38–0.45 μm at Day 14), likely due to structural alterations in saponins during its high-temperature extraction (80 °C), which compromised interfacial film integrity. SES emulsions (76.9 % purity) at 3.0–5.0 % showed moderate initial droplet sizes (d_3,2_ = 0.27–0.30 μm), which grew to 0.32–0.54 μm after 14 days (Fig. 5D), while UAA-APS (50.1 % purity) failed to stabilize emulsions beyond Day 3 (data no shown). The formation of smaller droplets may be attributed to efficient HLB-mediated interfacial packing, wherein the high purity of UATPPS enhances the ability of its saponins to rapidly form cohesive films with optimal hydrophilic-lipophilic balance. Unlike TPPS and SES systems, UATPPS emulsions maintained stable d_3,2_ values (<0.20 μm) across its full operational concentration range (1.5–5.0 %) throughout the 14-day storage period (Fig. 5D), demonstrating its unique resilience against coalescence. Furthermore, no significant changes in droplet size were observed during 14-day storage (Fig. 5D). Collectively, these findings demonstrate that the saponin extract obtained from COSC using UATPP-AP constitutes an excellent natural emulsifier, suitable for application in various emulsion-based products.

#### 3.5.5 In vitro antioxidant activity

Anti-oxidation activities of UATPPS, SES, and tea saponins standard were compared by in vitro antioxidant activity determining assays. DPPH assay measures protonradical scavenging activity of a tested sample, and a lower IC_50_ values related to higher antioxidant activity. For reference, vitamin C was used as a standard. As presented in the Fig. S2, the saponins samples demonstrated a dose-dependent free radical scavenging activity within the concentration range of 0.5 to 4.0 g/L, indicating a strong correlation between antioxidant performance and saponin concentration. Consequently, it is reasonable to conclude that saponins are the primary contributors to the observed antioxidant activity in the extracts. The IC_50_ of UATPPS, SES, tea saponins standard (TSS) and vitamini C (VC) were 1.23 ± 0.061, 1.971 ± 0.093, 1.783 ± 0.021 and 0.211 ± 0.016 g/L, respectively. The result showed that UATPPS possessed relatively stronger scavenging ability on DPPH radical than SES and tea saponins standard. The only difference between UATPPS and SES was the extraction method. Therefore, the higher free radical scavenging activity of UATPPS than that of SES indicated that the UATPP-AP method was more conducive to the enrichment of tea saponins with stronger hydrogen donation capacity than the traditional method. Notably, this performance surpasses that reported for saponins extracted from *Camellia oleifera* cake under optimized conditions (IC_50_ = 3.866 g/L) (Hu et al., 2012), highlighting the superior antioxidant capacity of UATPPS achieved through the UATPP-AP method.

In line with the DPPH assay, results from the TEAC and FRAP assays revealed that the TEAC and FRAP values of UATPPS were 242.37 ± 1.89 mg Trolox/g extract and 22.56 ± 0.23 μmol Fe^2+^/g extract, which were significantly larger than that of SES (186. 4 ± 2.77 mg Trolox/g extract and 14.67 ± 0.48 μmol Fe^2+^/g extract, respectively) and tea saponins standard (203.66 ± 1.42 mg Trolox/g standard sample and 18.32 ± 0.34 μmol Fe^2+^/g standard sample, respectively). These results clearly established the possibility that tea saponins extracted from COSC by UATPP-AP could be effectively employed as ingredient in health or functional food, to alleviate oxidative stress.

The superior antioxidant activity of UATPPS is closely tied to its structural preservation and interfacial functionality. FTIR analysis confirmed intact hydroxyl groups (3409 cm^−1^) and β-glycosidic bonds (841 cm^−1^), while HPLC data revealed enriched bidesmosidic saponins (E1/E2) (Fig. 4A-B), both critical for radical scavenging (Wang et al., 2021). The nano-scale emulsion droplets (d_3,2_ < 0.20 μm, Fig. 5D) stabilized by UATPPS form a cohesive interfacial layer, which may synergistically limit oxidative propagation by reducing reactive oxygen penetration. This potential physical-chemical synergy—stemming from high-purity saponins and emulsion stability—positions UATPPS as a multifunctional antioxidant system for lipid-rich matrices.

#### 3.5.6 In vitro antibacterial activity

**Fig. 6:**
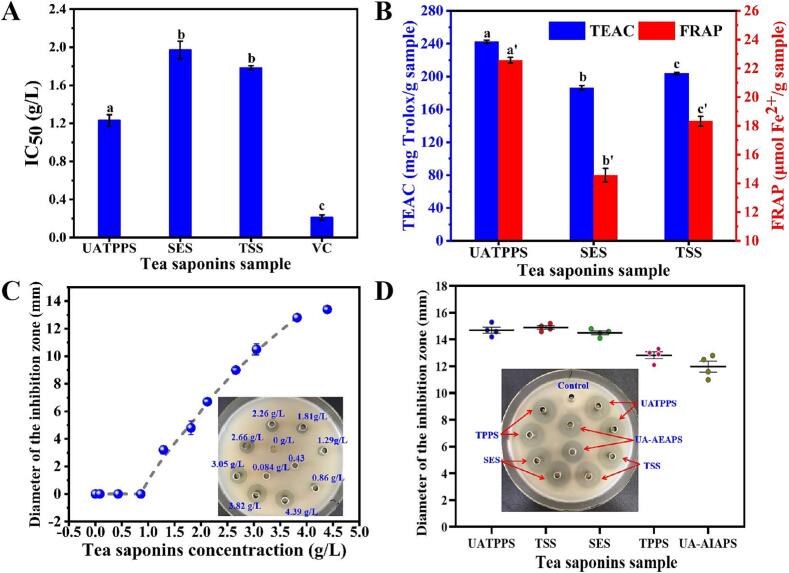
Antioxidant capacity and antibacterial activity of extracts. (A) Comparison of DPPH free radical scavenging activity; (B) Comparison of TEAC and FRAP activity; (C) Antibacterial effect of UATPPS on *E. coli* at different concentrations; (D) Comparison of the growth inhibitory effects of UATPPS, tea saponins standard and tea saponins extracted via other methods. Abbreviations of UATPPS, SES, TPPS and UAA-APS represent saponin extracts obtained using UATPP-AP, conventional technique SE-ACP, ATPP-AP and UAA-AP, respectively. TSS, tea saponins standard; VC, vitamin C. Different lowercase letters (e.g., a, b) for extraction yield and lowercase letters with primes (e.g., a′, b′) for saponin purity indicate statistically significant differences within the same group (*P* < 0.05).

It is reported that nonionic surfactants, characterized by the hydrophilic and hydrophobic groups, are easily interacting with cell membrane to change the membrane permeability and lead to cell death (Sharma et al., 2022). Saponins are typical nonionic surfactants, and previous studies have found that saponins are effective against a wide range of both gram-positive and gram-negative bacteria (Djamalladine et al., 2023; Fink & Filip, 2022). The potential of UATPPS to inhibit the growth of bacteria was also evaluated in this study. As shown in Fig. 6C, UATPPS inhibited the growth of gram-negative *E. coli* with concentrations above than 1.29 g/L; also the antibacterial activity was dose dependent, i.e., increasing with higher concentrations of the tea saponins in a certain range. The bacteriostatic circles of tea saponins standard and extracts obtained by reference methods were shown in Fig. 6D. It was observed that the growth inhibition zones of UATPPS showed no significant different with standard sample and SES. The results demonstrated that the tea saponin extracted using the UATPP-AP method exhibited antibacterial activity comparable to that obtained through conventional technique. The result also showed that UATPPS possessed stronger antibacterial activity than TPPS and UA-AIAPS. The extraction method for TPPS does not include ultrasonic treatment, potentially leading to a lower yield of tea saponins; the UAA-APS method lacks the TPP system purification step present in UATPPS, which may result in lower saponins purity in the extract. These findings further highlight the advantages of employing the UATPP-AP method for extracting tea saponins from *C.oleifera* cake.

The observed antibacterial activity of UATPPS may be synergistically supported by its emulsification properties. The formation of smaller emulsion droplets (d_3,2_ < 0.20 μm, Fig. 5D) in UATPPS-stabilized systems could increase bacterial contact probability through enhanced interfacial surface area compared to SES (Fig. 5C). This geometric advantage, combined with the preserved hydrophobic aglycone structures (methyl/methylene groups at 2928 cm^−1^, Fig. 4A), might promote membrane disruption efficiency. Additionally, the stability of UATPPS emulsions over 14 days (Fig. 5D) suggests potential for sustained saponin release at oil-water interfaces, which could maintain antimicrobial activity in emulsified systems. While the exact mechanistic interplay requires further investigation, these findings imply that the UATPP-AP method optimizes both structural integrity and interfacial functionality, potentially enhancing the practical utility of tea saponins in preservation-sensitive formulations.

## 4 Conclusion

This study develops a two-stage alkaline-driven extraction technology (UATPP-AP) that synergizes ultrasound-assisted three-phase partitioning with acid precipitation for sustainable saponin recovery from *C. oleifera* seed cake. By optimizing the aqueous phase to pH 10 during *t*-butanol/ammonium sulfate partitioning, the system leverages alkaline-enhanced saponins solubility to achieve 13.5 g/100 g yield within 30 min (92 % faster than Soxhlet extraction), while concurrently removing interfacial impurities and recovering 91 % *t*-butanol through five operational cycles. Subsequent pH reduction to 4.5 induces selective saponins precipitation, eliminating acetone dependency and achieving 85 % purity (vs. 70 % conventional method), with intact oleanane-type triterpene structures verified by FTIR and HPLC-ELSD analyses. Scale-up validation in a 30 L reactor (actual working volume 22.5–25 L) demonstrates industrial viability (12.7 g/100 g yield, 82 % purity), producing multifunctional saponins with superior interfacial activity (5.21 mN/m), 14-day emulsion stability, and significant dual bioactivities (antioxidant IC_50_ 1.23 g/L; antimicrobial MIC 1.29 g/L). The technology establishes a green paradigm for oilseed waste valorization through pH-engineered mass transfer enhancement and closed-loop *t*-butanol recirculation, providing a scalable route to produce natural emulsifiers and bioactive agents with minimized organic solvent input. However, this study has two limitations: pilot-scale validation in 30 L reactors requires industrial-scale amplification (e.g., hectoliter systems) to verify mass transfer efficiency and energy consumption; additionally, quantitative analysis of process economics (e.g., solvent recycling costs and equipment payback periods) needs further refinement.

## CRediT authorship contribution statement

**Zhihong Chen:** Writing – original draft, Methodology, Funding acquisition, Conceptualization. **Jinlin Fan:** Visualization, Supervision, Investigation. **Chao Zhao:** Writing – review & editing, Supervision. **Taoyuan Huang:** Methodology. **Zhiying Guo:** Software, Methodology. **Changyang Qiu:** Writing – review & editing, Project administration, Funding acquisition. **Jiacong Deng:** Writing – review & editing, Project administration, Funding acquisition.

## Funding

This work was supported financially by the high-level personnel research funding of Fujian Polytechnic Normal University (No. 404056), and 2023 Science and Technology Innovation Project of Department of Forestry of Jiangxi Province, China (Innovation Project (2023) No. 17).

## Declaration of competing interest

The authors declare that they have no known competing financial interests or personal relationships that could have appeared to influence the work reported in this paper.

## Data Availability

Data will be made available on request.

## Appendix A Supplementary data

Supplementary material Image 1
