# Supplementary material for: Integrated pH-responsive three-phase system: Time- and solvent-efficient biorefining of Camellia oleifera waste into multifunctional saponins
Source: Food Chem X. 2025 Aug 5;29:102870. doi: 10.1016/j.fochx.2025.102870 (PMC12355557; doi:10.1016/j.fochx.2025.102870)
Supplement: Supplementary file 1 — Supplementary material [file mmc1.docx]

**Integrated pH-responsive three-phase system: Time- and solvent-efficient biorefining of *Camellia Oleifera* waste into multifunctional saponins**

Zhihong Chen^a^, Jinlin Fan^a^, Chao Zhao^c^, Taoyuan Huang^a^, Zhiying Guo^a^, Changyang Qiu^b,*^ Jiacong Deng^a,*^

^a^College of Food and Bioengineering, Fujian Polytechnic Normal University, Fuqing 350300, China

^b^ Institute of Jiangxi Oil-tea Camellia, Jiujiang University, Jiujiang 332005, China

^c^ College of Marine Sciences, Fujian Agriculture and Forestry University, Fuzhou 350117, China

^*^ Corresponding author:

E-mail addresses: 310222649@qq.com(C.Qiu); dengjc810508@163.com(J.Deng)


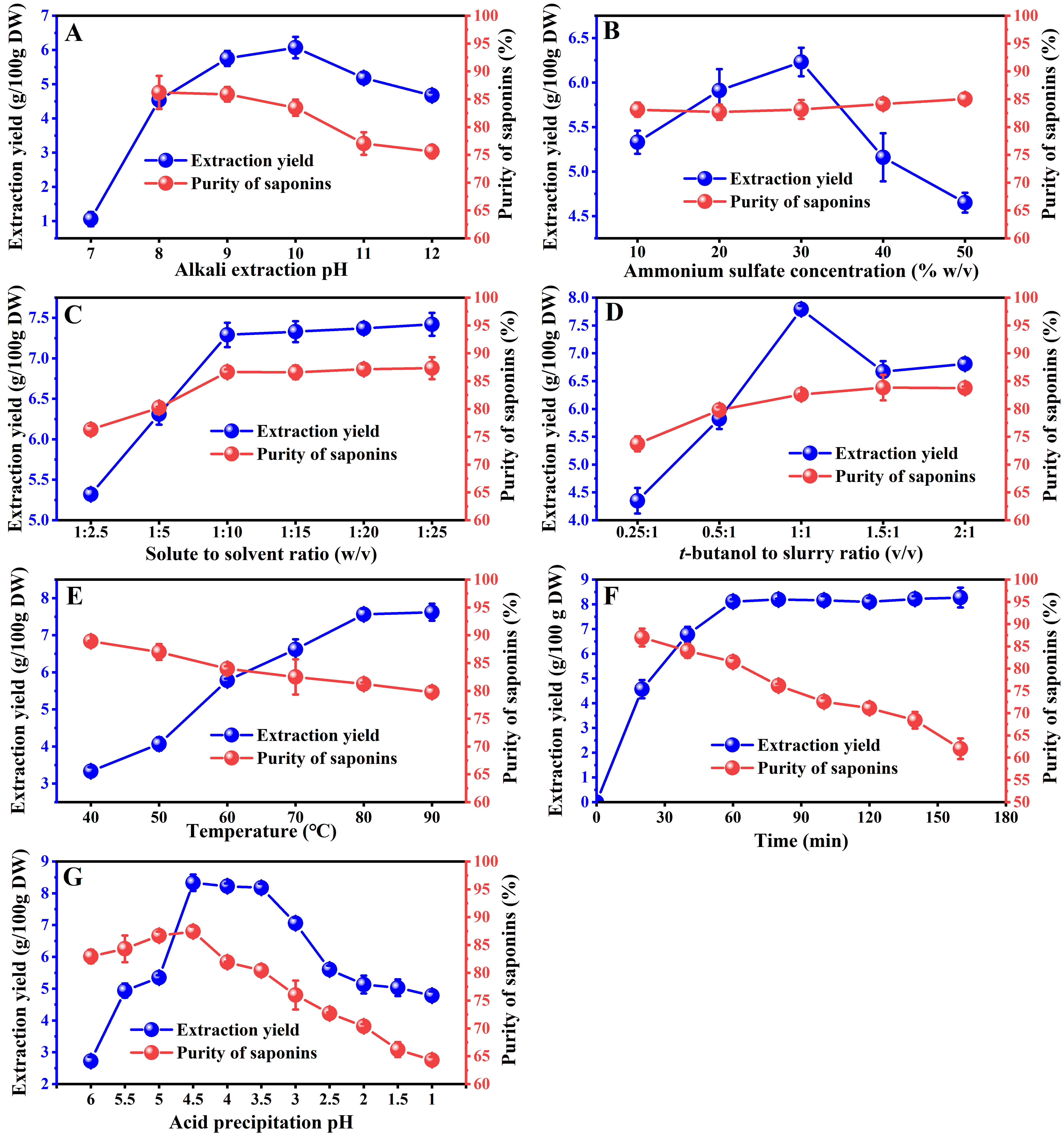


**Fig. S1** The optimal conditions for extracting tea saponins from *Camellia oleifera* seed cake using the TPP-AP method are: alkaline extraction at pH 10 (A), 30% (w/v) ammonium sulfate concentration (B), solute-to-solvent ratio 1:10 (w/v) (C), *t*-butanol-to-slurry ratio 1:1 (v/v) (D), 80℃ (E) for 60 min (F), and acid precipitation at pH 4.5 (G)


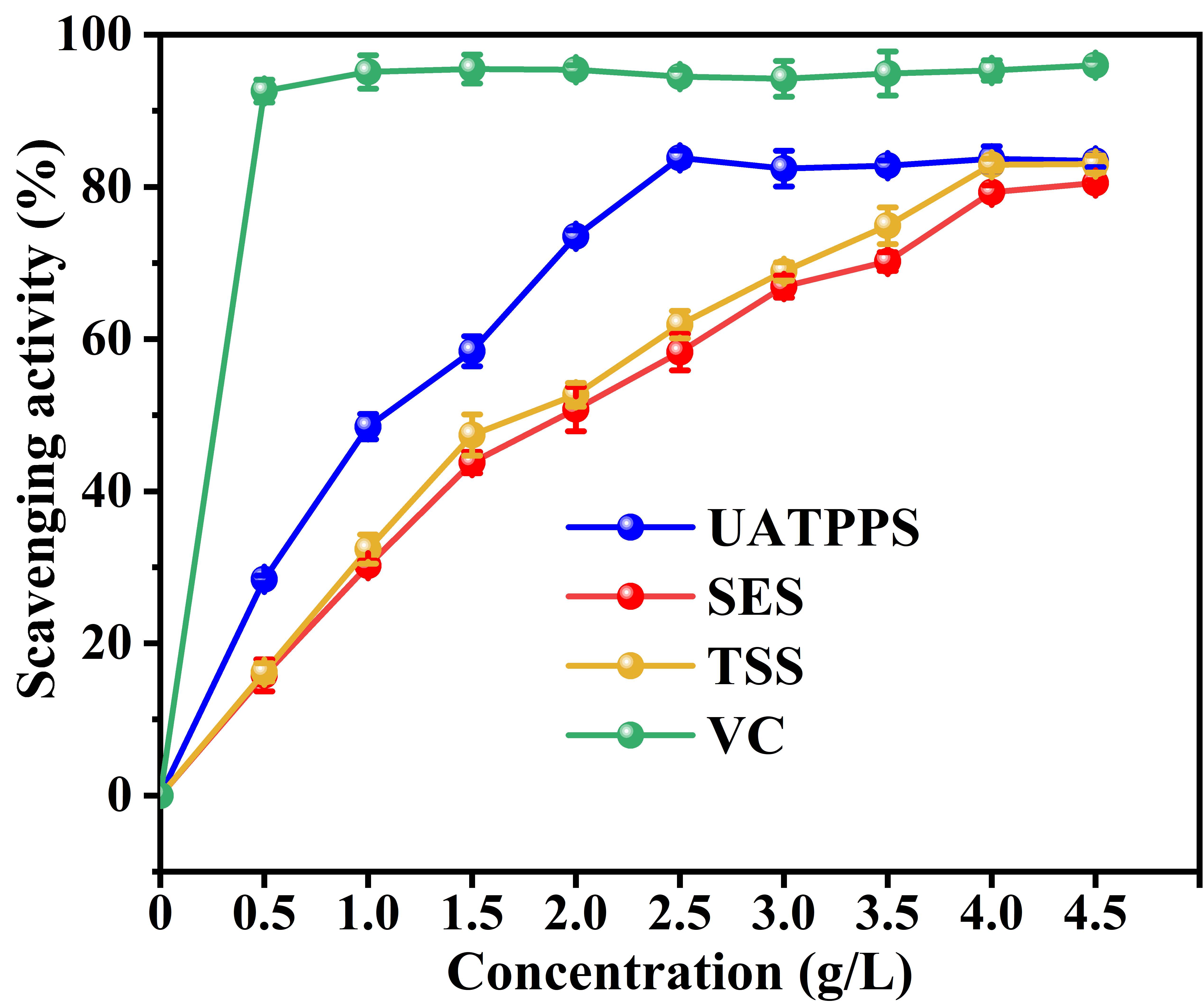


**Fig. S2** Scavenging effects of test samples on DPPH free radical. Abbreviations of UATPPS and SES represent saponin extracts obtained using UATPP-AP and conventional technique SE-ACP, respectively. TSS, tea saponins standard; VC, vitamin C.
